# The pubovesical complex-sparing technique on laparoscopic radical prostatectomy

**DOI:** 10.1590/S1677-5538.IBJU.2017.0359

**Published:** 2018

**Authors:** Rafael Batista Rebouças, Rodrigo Campos Monteiro, João Paulo Pereira Lima, Filipe de Pádua B. F. Almeida, Cesar Araujo Britto, Marcos Tobias Machado, Carlo Passerotti

**Affiliations:** 1 Departamento de Urologia, Hospital da Polícia Militar Edson Ramalho, João Pessoa, PB, Brasil; 2 Departamento de Urologia, Universidade de João Pessoa - UNIPE, João Pessoa, PB, Brasil; 3 Departamento de Uro-oncológica, Hospital São Vicente de Paulo, João Pessoa, PB, Brasil; 4 Departamento de Urologia, Hospital Universitário Onofre Lopes, Universidade Federal do Rio Grande do Norte, Natal, RN, Brasil; 5 Faculdade de Medicina do ABC, Santo André, SP, Brasil; 6 Centro de Cirurgia Robótica, Hospital Alemão Oswaldo Cruz, São Paulo, SP, Brasil; 7 Departamento de Urologia, Escola de Medicina de São Paulo - Laboratório de Investigação Médica (LIM55), São Paulo, SP, Brasil

## Abstract

**Introduction:**

Preservation of urinary continence is a great challenge in Radical Prostatectomy. In order to improve functional results, Asimakopoulos et al. (2010) described a robot-assisted surgical technique with preservation of the pubovesical complex (PVC). We present a pure laparoscopic execution.

**Presentation:**

A 61-year-old male patient with a diagnosis of prostate cancer, with PSA 6.54ng/ml, DRE: T1C and Gleason 6 (3+3) 1/12 fragments. All therapeutic possibilities were discussed, including active surveillance. The patient opted for surgical treatment.

A transperitoneal technique was used. We started the dissection on the left side, in the limit between the detrusor and the base of the prostate. The left seminal vesicle was dissected and left neurovascular bundle released by a high anterior dissection. We repeated the same procedure on the right side. The urethra was then divided, prostatic apex was laterally drawn and PVC was released. The bladder neck was divided and an urethrovesical anastomosis was achieved. A pelvic drain was placed.

**Results:**

The total operative time was 150 minutes. The estimated blood loss was 300mL. The drain was removed on the 1st postoperative day and the patient was discharged. The Foley catheter was removed after 7 days and the patient remained completely dry. Hystopathology revealed adenocarcinoma Gleason 6, negative margins. PSA after 30 days was <0.04ng/mL, and the patient reported partial penile erection.

**Conclusion:**

The Pubovesical Complex-Sparing Technique on Laparoscopic Radical Prostatectomy was feasible and safe. Further adequately designed studies are needed to confirm whether this technique enhances early functional outcomes.

## ARTICLE INFO

**Available at: http://www.intbrazjurol.com.br/video-section/20170359_Reboucas_et_al**

**Int Braz J Urol. 2018; 44 (Video #12): 844-5**

